# Production of a new non-specific nuclease from *Yersinia enterocolitica* subsp. *palearctica*: optimization of induction conditions using response surface methodology

**DOI:** 10.1080/13102818.2014.915612

**Published:** 2014-08-26

**Authors:** Xiu-Juan Fang, Zhen-Xing Tang, Zhen-Hua Li, Zhi-Liang Zhang, Lu-E Shi

**Affiliations:** ^a^ College of Life and Environmental Sciences, Hangzhou Normal University, Hangzhou, Zhejiang, P.R. China; ^b^ Date Palm Research Center, King Faisal University, Al-hasa, Saudi Arabia; ^c^ Department of Food Science, Anqing Vocational & Technical College, Anqing, Anhui, P.R. China

**Keywords:** *Y*. e*nterocolitica* subsp. *palearctica*, non-specific nuclease, induction conditions, response surface methodology

## Abstract

A new non-specific nuclease from *Yersinia enterocolitica* subsp. *palearctica* (*Y. NSN*) was expressed in *Escherichia coli* (*E. coli*) BL 21 Star^TM^ (DE3)plysS. Induction conditions, including isopropyl-β-D-thiogalactoside (IPTG) concentration, cell density (OD600), induction time and induction temperature, were optimized using response surface methodology. Statistical analysis of the results revealed that induction temperature and all the quadratic terms of variables had significant effects on enzyme activity of *Y. NSN*. The optimal induction conditions were as follows: 1.5 mmol/L IPTG, OD600 of 0.80, induction time of 20.5 h, and induction temperature of 32 °C. Under the optimized conditions, the highest enzyme activity could be obtained.

## Introduction

Recombinant proteins are widely used in various fields.[1] The *E. coli* expression system is extensively applied in the production of recombinant proteins because of its advantages such as the ease of handling, inexpensive growth requirements and high-level accumulation of target products.[1,2] Induction conditions are very important for the expression of recombinant proteins.[3] Many studies on the optimization of induction conditions for the production of recombinant proteins have been reported.[4,5] Of all induction conditions, inducers play an important role in the expression of recombinant proteins. Isopropyl-β-D-thiogalactoside (IPTG), a highly stable and effective inducer of the T7 promoter for the expression of target recombinant proteins, is widely used in laboratories.[6] Studies have been performed with the aim of optimizing IPTG concentration for the induction of *E. coli* in fed-batch cultures.[7,8] Whereas high induction temperature can promote cell growth, it can also result in a high probability of plasmid loss and stimulate mispartition of an expression vector.[9,10] Cell density (OD600) and induction time also play critical roles in achieving high protein yields.[10–12] Therefore, the optimal induction conditions for specific recombinant protein expression are highly needed.

Non-specific nucleases, with the ability to degrade both DNA and RNA, have been isolated from many sources such as viruses, bacteria, fungi and animals.[13–16] Non-specific nucleases play very important roles in different aspects of biological process, including DNA replication, DNA repair and recombination of DNA and RNA processing, maturation and editing, host defence against foreign nucleic acid molecules, etc.[17–19] Non-specific nucleases, such as bovine pancreatic DNase I, staphylococcal nuclease and Serratia nuclease, are used in industrial biotechnology for processing of various pharmaceutical and biotechnological products. Certain amino acid sequences or three-dimensional structural preferences of these non-specific nucleases have been reported.[20,21] Up to date, one of the best-studied non-specific nuclease is the Serratia nuclease.[22–25] It is commercially available as benzonase, being used as a tool in industrial biotechnology for the removal of nucleic acids.

Bioprocess optimization through statistical design is a common practice in biotechnology fields and has proved to be a more useful tool as compared to the common ‘one-factor-at-a-time’ method,[10] which cannot provide information about the interaction of different effective variables and requires more experimental data-sets. Response surface methodology (RSM) can provide statistical models that help to understand the interaction of different variables and predict the optimized conditions.[1,26] There are a number of RSM designs such as central composite, Box–Behnken, three-level factorial, D-optimal, hybrid, pentagonal, hexagonal, etc. The use of RSM can allow rapid and economical determination of the optimized conditions with fewer experiments.

Our previous preliminary experiments show that *Yersinia enterocolitica* subsp. *palearctica* non-specific endonuclease (*Y. NSN*) can degrade both DNA and RNA in a sequence- or structure-independent manner. It can potentially be used more widely than the other existing non-specific nucleases. However, the nuclease activity is very low in the wide-type strain *Y. enterocolitica*. Thus, in this paper, the recombinant *Y. NSN* was expressed in *E. coli*. Induction conditions for the expression of *Y. NSN*, such as IPTG concentration, cell density (OD600), induction time and induction temperature, were optimized by RSM. To the best of our knowledge, this is the first report on the optimization of induction conditions of *Y. NSN* by RSM.

## Materials and methods

### Bacterial strains and expression vectors

*E. coli* host strains BL21, BL21 (DE3)pLysS and BL 21 Star^TM^ (DE3)plysS (Invitrogen) were used for gene expression experiments. Host strain DH5α (Invitrogen) was used as both expression and cloning strains. Vectors pET-24a and pET-24d (Invitrogen) were used for cloning and expression studies.

The *Y. enterocolitica* strain was isolated from the stool of a human patient. The protocol was according to Bhaduri and Wesley [27] with some modification. Fresh faecal sample was collected from the local hospital. The interval from sample collection to sample analysis in the laboratory was between 48 and 72 h. One gram from faecal sample was suspended in 9.0 mL of 0.1% peptone water and mixed in a blender for 30 s. One millilitre of the suspension was added to 9.0 mL of irgasan–ticarcillin–potassium chlorate broth in a tube and vortexed. The enrichment was held at room temperature for 48 h. Selectively enriched sample was vortexed and diluted 1:10 in 0.1% peptone water, and a 100 μL aliquot was plated on cefsulodin–irgasan–novobiocin agar and incubated at 30 °C for 24 h. *Y. enterocolitica* colony with a deep red centre was isolated. The identification of this strain was through the analysis of 16s rRNA gene sequencing.

### Culture media

Luria-Bertani medium without 50 μg/mL of kanamycin was used for the culture. The defined medium contained: 10 g of tryptone, 5 g of yeast extract and 10 g of NaCl. All chemicals were obtained from Sigma-Aldrich.

### Construction of expression vectors

The 783 bp coding region of *Y. NSN* (6 × His-tagged) from cDNA with peptide signal cutting was amplified by the polymerase chain reaction (PCR) method. The forward primer was 5′-TTAATTATTCATATGTCC GCGCCCAAAACC-3′. And the reverse primer was 5′-AATATACTCGAGATCGCATCCAATTGT-3′. PCR was performed in a 30-μL reaction mix containing 50 mmol/L of KCl, 10 mmol/L of Tris-HCl (pH 8.3), 1.5 mmol/L of MgCl_2_, 100 μg/mL of gelatin, 0.2 mmol/L of dNTPs, 1.25 U of DNA polymerase (New England Biolabs) and 50 pmol of each forward and reverse primer. The thermocycling parameters used for PCR were as follows: 1 min at 60 °C for annealing; 2 min at 72 °C for extension; and 1 min at 95 °C for denaturation. After 30 cycles, amplified cDNA products were digested and cloned into pET-24a vector, and finally, this engineered vector was transformed into expression host. Furthermore, the whole 852 bp DNA fragment coding region of *Y. NSN* (6 × His-tagged) from cDNA was also amplified and cloned into pET-24d vector. The detailed protocol was similar with that for the amplification and cloning of the 783 bp coding region of *Y. NSN* described above.

### Choice of expression host and vectors of *Y. NSN*

In order to optimize the production of *Y. NSN* in *E. coli*, different vectors (pET-24a and pET-24d) and expression hosts (DH5α, BL21, BL21 (DE3)pLysS and BL21 Star^TM^ (DE3)plysS) were tested. Each strain cells transformed with pET-24a or pET-24d were grown at mid-log phase (OD600 of 0.6), while expression of *Y. NSN* was induced with 1.0 mmol/L of IPTG at 37 °C. The cells were harvested by centrifugation at 4 °C, 8000 *g* for 10 min, and resuspended in 20 mmol/L of Tris-HCl buffer (pH 7.0) supplemented with 20 mmol/L of MgCl_2_. Afterwards, the supernatant of cell lysis (three cycles of freezing −20 °C and thawing 50 °C) was kept for the analysis of enzyme activities.

### Optimization of induction conditions

RSM was applied to optimize the induction conditions for *Y. NSN* production. A 2^4^ full-factorial central composite rotary design for four independent variables each at five levels was employed to fit a second-order polynomial model, which indicated that 30 experiments were required for this procedure.[28] The software package Design Expert version 7.0 (Stat-Ease Inc., Minneapolis, MN, USA) was used to obtain the interactive effect of four variables. The coded and uncoded variables are listed in Table 1. The variables were cell density OD600 (0.6, 0.7, 0.8, 0.9 and 1.0), induction temperature (28, 31, 34, 37 and 40 °C), IPTG concentration (0.5, 1.0, 1.5, 2.0 and 2.5 mmol/L) and induction time (16, 18, 20, 22 and 24 h). Table 2 illustrates the central composite design at the given range of the aforementioned parameters in terms of codes and actual terms.

**Table 1.** Coded and non-coded levels of independent variables used in the RSM design.

	Levels				
Independent variables	−2	−1	0	1	2
*A*:OD600 (nm)	0.6	0.7	0.8	0.9	1.0
*B*: induction temperature (°C)	28	31	34	37	40
*C*: IPTG concentration (mmol/L)	0.5	1	1.5	2	2.5
*D*: induction time (h)	16	18	20	22	24

**Table 2.** Response values for relative enzyme activity obtained from RSM.

	Coded values				Actual values				
Run	*A*	*B*	*C*	*D*	*A*	*B*	*C*	*D*	Response values (relative enzyme activity)
1	−1	1	1	1	0.7	37	2	22	48
2	2	0	0	0	1.0	34	1.5	20	75
3	0	0	−2	0	0.8	34	0.5	20	75
4	0	0	0	0	0.8	34	1.5	20	100
5	0	0	0	−2	0.8	34	1.5	16	87
6	1	1	1	−1	0.9	37	1.5	18	45
7	0	0	0	0	0.8	34	1.5	20	100
8	−1	1	1	−1	0.7	37	2	18	60
9	1	−1	−1	1	0.9	31	1	22	85
10	0	0	2	0	0.8	34	2.5	20	68
11	0	2	0	0	0.8	40	1.5	20	38
12	−1	1	−1	−1	0.7	37	1	18	72
13	−1	−1	1	1	0.7	31	2	22	85
14	0	0	0	2	0.8	34	1.5	24	84
15	0	−2	0	0	0.8	28	1.5	20	93
16	0	0	0	0	0.8	34	1.5	20	100
17	1	1	1	1	0.9	37	2	22	50
18	0	0	0	0	0.8	34	1.5	20	100
19	1	−1	1	−1	0.9	31	2	18	73
20	1	−1	−1	−1	0.9	31	1.0	18	77
21	1	−1	1	1	0.9	31	2	22	86
22	1	1	−1	1	0.9	37	1.0	22	42
23	−1	−1	1	−1	0.7	31	2	18	78
24	−1	1	−1	1	0.7	37	1.0	22	50
25	1	1	−1	−1	0.9	37	1.0	18	60
26	0	0	0	0	0.8	34	1.5	20	90
27	−1	−1	−1	−1	0.7	31	1.0	18	82
28	−2	0	0	0	0.6	34	1.5	20	85
29	−1	−1	−1	1	0.7	31	1.0	22	82
30	0	0	0	0	0.8	34	1.5	20	100

Relative enzyme activity was taken as the response which was assumed to be influenced by four variables. Thus, the experimental results (Table 2) were analysed by multiple regressions through the least-square method. A second-order polynomial equation was used to express the response as a function of the independent variables as follows:(1) 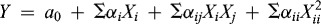
where *Y* represents the measured response; *a*_0_ is a constant; α*_i_*, α*_ij_* and α*_ii_* are the linear, quadratic and interactive coefficients of the model, respectively; and *X_i_* and *X_j_* are the levels of the independent variables.

The second-order polynomial coefficients were calculated to estimate the response of the dependent variable. Response surface plots were also drawn. Analysis of variance (ANOVA) was performed in order to fit the second-order polynomial equations for all response variables. The significance of the model equation and model terms were evaluated by *F*-test, while the quality of fit of the polynomial equations were expressed by the coefficient of determination (*R* ^2^) and adjusted *R* ^2^.[7] The combination of different optimized parameters, which gave maximum response (relative enzyme activity), was tested experimentally to see the viability of the model. The test of statistical significance was based on the total error criteria with a confidence level of 95.0%.

### Enzyme activity analysis

Enzyme activity was determined according to the degree of DNA degradation. Approximately 2.0 μL of diluted enzyme solution was incubated in 40-μL DNA of calf thymus (100 ng/μL) as a substrate. After incubation at 37 °C for 5 min, the reaction was stopped by adding 8.0 μL of 6 × DNA loading buffer (10 mmol/L Tris-HCl, 60 mmol/L EDTA, 40% sucrose, 0.05% bromophenol blue and pH 7.6). The reaction products were then subjected to 1.0% agarose gel electrophoresis. The degraded degree of nucleic acid could be visualized and calculated by staining with 0.25 μg/mL of ethidium bromide.

## Results and discussion

### Choice of host for *Y. NSN* gene expression

Varying several parameters such as the bacterial host and vector could affect the expression of recombinant proteins. In the present study, the evaluation of different expression hosts including DH5α, BL21, BL21 (DE3) pLysS and BL 21 Star^TM^ (DE3)plysS and vectors including pET-24a and pET-24d were performed. The results (not shown) revealed that protein could only be well expressed in BL 21 Star^TM^ (DE3)plysS host strain using pET-24a as a vector. When other expression host was used, the expression level could only reach 20% of that in BL21 Star^TM^ (DE3)plysS host strain (results not shown). The difference of the expression level of *Y. NSN* in the host might be related to the toxicity of the nuclease to the host. Therefore, BL21 Star^TM^ (DE3)plysS and pET-24a were chosen as the expression host and vector for the optimization of the production of *Y. NSN*, respectively.

### Optimization of induction conditions of *Y. NSN* expression

#### Fitting the model

To minimize the experimental runs and time for optimizing induction conditions, an experimental design was adopted on the basis of RSM.[29] The levels of these independent variables were determined based on preliminary experiments. The experimental values from 30 simplified experimental runs are listed in Table 2. The ANOVA for relative enzyme activity is shown in Table 3. For any of the terms in the model, a large regression coefficient and a small *p*-value would indicate a more significant effect on the response variables.[29,30] Thus, the variables having the significant effect on enzyme activities were induction temperature (*B*), the interaction term between induction temperature (*B*) and induction time (*D*), and the quadratic terms of OD600 (*A*), induction temperature (*B*), IPTG concentration (*C*) and induction time (*D*).

**Table 3.** Results of the analysis of variance to response surface quadratic model.

Source	Sum of squares	Degree of freedom	Mean square	*F*-value	*p*-value
Model	9745.08	14	696.08	20.02	<0.0001
*A*: OD600	145.04	1	145.04	4.17	0.0591
*B*: temperature	4565.04	1	4565.04	131.28	<0.0001
*C*: IPTG	63.38	1	63.38	1.82	0.1970
*D*: induction time	26.04	1	26.04	0.75	0.4004
*AB*	45.56	1	45.56	1.31	0.2703
*AC*	1.56	1	1.56	0.045	0.8350
*AD*	76.56	1	76.56	2.20	0.1586
*BC*	18.06	1	18.06	0.52	0.4822
*BD*	351.56	1	351.56	10.11	0.0062
*CD*	126.56	1	126.56	3.64	0.0757
*A*^2^	984.00	1	984.00	28.30	<0.0001
*B*^2^	2535.50	1	2535.50	72.92	<0.0001
*C*^2^	1806.07	1	1806.07	51.94	<0.0001
*D*^2^	584.07	1	584.07	16.80	0.0009
Residual	521.58	15	34.77		
Lack of fit	438.25	10	42.82	2.63	0.1488
Pure error	83.33	5	16.67		
Total	10,266.67	29			

*R* ^2^ = 0.9492, adjusted *R* ^2^ = 0.9018 and coefficient of variation (CV) = 7.79%

In order to estimate the optimal enzyme activity, the full quadratic second-order polynomial equation was fitted by applying multiple regression analysis on the experimental data (Table 2):(2) 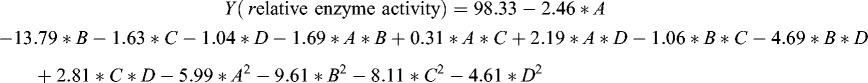



**Figure 1.**
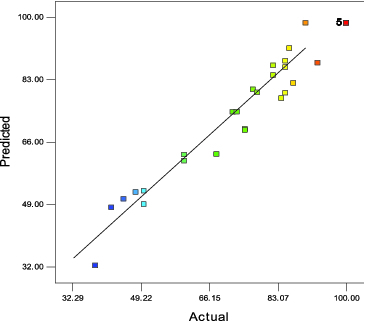
Predicted and experimental values for relative enzyme activities (*R* ^2^ = 0.949).

As shown in Table 3, the model was highly significant (*p* < 0.01). The resultant second-order polynomial model adequately represented the experimental data (*R* ^2^ = 0.949), which could explain 94.9% of the enzyme activity variability. Moreover, the low coefficient of variation (CV) value of 7.79% illustrates the great degree of precision with which the actual values were compared. Lack of fit analysis was insignificant (*p* > 0.05), which indicates that the model fitted the experimental data very well. Figure 1 is a plot of the predicted versus experimental values of enzyme activities, and it also shows that the two sets of data agreed with each other quite well.

#### Analysis of response surfaces

**Figure 2.**
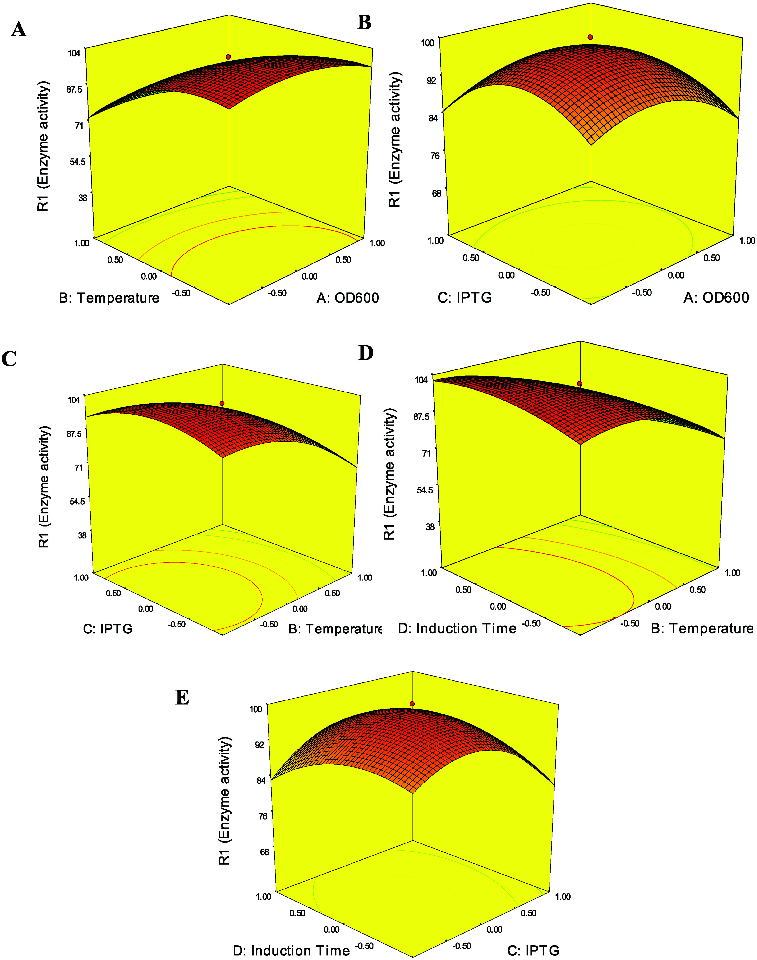
Response surface plots showing the effect of interaction factors on enzyme activity: (A) OD600 and induction temperature (IPTG concentration 1.5 mmol, induction time 20 h); (B) OD600 and IPTG concentration (induction temperature 34 °C, induction time 20 h); (C) IPTG concentration and induction temperature (OD600 0.8, induction time 20 h); (D) induction temperature and induction time (IPTG concentration 1.5 mmol, induction temperature 34 °C); and (E) induction time and IPTG concentration (OD600 0.8, induction temperature 34 °C).

In order to illustrate the effect of the independent variables on enzyme activities, surface responses were constructed by varying two variables within the experimental ranges and holding the other variables at the central point. The response surface curves are given in Figure 2.

As shown in Table 2, induction temperature had a significant effect on enzyme activity (*p* < 0.001). This result can also be easily observed in Figure 2(A), 2(C) and 2(D). Low induction temperature had a positive effect on enzyme activity. This result can be explained by protein accumulation at low induction temperature process in recombinant bacteria. Therefore, by simply lowering the induction temperature, production of active recombinant enzyme in bacteria cells would be enhanced. However, there was a negative quadratic effect at high temperature (Equation 2), i.e. higher temperature would reduce the enzyme activity of *Y. NSN*. The high induction temperature was detrimental to enzyme expression, probably because the higher specific growth rates placed a much higher metabolic burden on the cell and promoted inclusion bodies to be formed.[6,31] Pan et al. [6] optimized culture conditions for the production of *cis*-eoxysuccinate hydrolase (CESH) by RSM. The results showed that induction temperature significantly influenced the production of CESH and production of CESH could be enhanced by the control of induction temperature. Similar results can also be found in the work of Lo et al. [32]

IPTG concentration should be optimized because of its great contribution to *Y. NSN* expression. Low enzyme activity could be obtained when IPTG concentration is low (Figure 2(B), 2(C) and 2(E)). However, enzyme activity was reduced at high IPTG concentration. This could also be reflected by the negative quadratic term (C) at high concentration (Equation 2). Many similar studies have been reported. Wang et al. [10] found that higher IPTG concentrations did not significantly improve the hk2a fusion protein levels. Tabandeh et al. [33] optimized the induction conditions of recombinant interferon beta by RSM. Their results indicated that the production of recombinant interferon beta could be inhibited under high IPTG concentration. In our present study, inhibitory effects of high IPTG concentration on enzyme activity were also found. High concentration of IPTG can inhibit bacterial growth because the change in the metabolic pathway from cell growth to the production of heterologous protein happens rapidly at high IPTG concentration.[33,34] On the other hand, it seems that the expression level of recombinant protein is increased by using higher concentrations of IPTG. However, the results (Equation 2) showed a negative quadratic effect on enzyme activity at high IPTG concentration, thus indicating that a middle point of IPTG concentration is suitable for obtaining a high level of enzyme activity.

There are different optimized induction times for the expression of different recombinant proteins. The optimized induction time for heat-stable alkaline protease from B. *stearothermophilus* is 72 h,[35] or 6 h for human BD4,[36] and 24 h for *E. coli* SVP2.[1] As shown in Figure 2(D) and 2(E), enzyme activity of *Y. NSN* was lower with shorter induction time. However, longer induction time could result in lower enzyme activity. The reason may be cell lysis and proteolytic degradation.[10,11] Similar with our study, some other previous reports propose that the long induction time also had a prominently negative effect on protein expression.[1,37] Kang et al. [3] optimized vascular endothelial growth factor_165_ (rhVEGF_165_) expression in *E. coli* and showed that shorter or longer induction time would decrease the expression level of rhVEGF_165_. The assumed reason probably was intracellular expression of the target protein which did not need to undergo a complicated translocation process.[1]

Another factor that is well known to affect recombinant protein expression is cell density.[4] In our study, low OD600 had a positive effect on enzyme activity (Figure 2(A) and 2(B)). However, there was a negative quadratic effect at high OD600 (Equation 2). Thus, enzyme activity would be decreased at high OD600. Some similar studies have been reported. Wang et al. [10] found that the cells induced in the early log phase promoted the concentration of the expressed protein. Papaneophytou and Kontopidis [28] found the cell density (OD600) was an important factor for the production of TNF-α in a soluble form. TNF-α production was increased with the increasing of OD600 up to a certain point and then decreased.

Interactions between the induction conditions were predicted by RSM, and the results are shown in Table 3. In our study, the interactions between induction temperature and induction time had a significant effect on enzyme activity (Figure 2(D), Equation 2). Enzyme activity was higher with low induction temperature and longer induction time. In contrast, the higher induction temperature and shorter induction time could result in lower enzyme activity. Pan et al. [6] reported similar results that only the interaction between induction temperature and induction time was significant. However, Lo et al. [32] observed that the interactions between the culture conditions did not exert a significant effect on enzyme activity. Because the quadratic model (Equation 2 and Table 3) was checked to be adequate, our results showed that induction conditions could influence enzyme activity in the expression system.

#### Optimization of induction conditions

The optimal parameters obtained by RSM were as follows: OD600 (0.80), induction temperature (32 °C), IPTG concentration (1.5 mmol/L) and induction time (20.5 h). Under these optimal conditions, up to 100% relative enzyme activity could be reached.

## Conclusions

*Y. NSN* was successfully expressed in *E. coli*. The obtained results showed that a second-order polynomial model from RSM could be used to optimize induction conditions of *Y. NSN*. Induction temperature and all quadratic terms of investigated variables had significant effects on enzyme activity of *Y. NSN*. RSM is an effective technique for analysing and optimizing the induction conditions. This study will facilitate the production and application of this *Y. NSN*.
